# Physical Activity Enhances Metabolic Fitness Independently of Cardiorespiratory Fitness in Marathon Runners

**DOI:** 10.1155/2015/806418

**Published:** 2015-03-03

**Authors:** M. J. Laye, M. B. Nielsen, L. S. Hansen, T. Knudsen, B. K. Pedersen

**Affiliations:** ^1^Centre of Inflammation and Metabolism, Rigshospitalet, Department of Biomedical Sciences, University of Copenhagen, 2200 Copenhagen, Denmark; ^2^The Buck Center for Research on Aging, Novato, CA 94945, USA

## Abstract

High levels of cardiovascular fitness (CRF) and physical activity (PA) are associated with decreased mortality and risk to develop metabolic diseases. The independent contributions of CRF and PA to metabolic disease risk factors are unknown. We tested the hypothesis that runners who run consistently >50 km/wk and/or >2 marathons/yr for the last 5 years have superior metabolic fitness compared to matched sedentary subjects (CRF, age, gender, and BMI). Case-control recruitment of 31 pairs of runner-sedentary subjects identified 10 matched pairs with similar VO_2max_ (mL/min/kg) (similar-VO_2max_). The similar-VO_2max_ group was compared with a group of age, gender, and BMI matched pairs who had the largest difference in VO_2max_ (different-VO_2max_). Primary outcomes that defined metabolic fitness including insulin response to an oral glucose tolerance test, fasting lipids, and fasting insulin were superior in runners versus sedentary controls despite similar VO_2max_. Furthermore, performance (velocity at VO_2max_, running economy), improved exercise metabolism (lactate threshold), and skeletal muscle levels of mitochondrial proteins were superior in runners versus sedentary controls with similar VO_2max_. In conclusion subjects with a high amount of PA have more positive metabolic health parameters independent of CRF. PA is thus a good marker against metabolic diseases.

## 1. Introduction

High levels of physical activity (PA) and cardiorespiratory fitness (CRF) are independently associated with a low risk for many chronic diseases [1] and mortality [2]. While related, the distinction between these CRF and PA is critical. CRF integrates many different physiological systems into a single measure of function. On the other hand PA is comprised of any body movements, which may not necessarily lead to improvements in CRF. However, in general metareviews and independent studies indicate that the protective effect of CRF is greater when compared to PA, in most [3–5], but not all cases [6]. One factor in the discrepancy between the relative health effects for CRF versus PA is the level of precision in measurements of CRF versus PA. Assessment of CRF in large epidemiological studies is typically assessed by a treadmill or ergometer test [3], while PA is assessed by a questionnaire [7]. Indeed, the correlation between reported PA using the international physical activity questionnaire and CRF as measured by a treadmill test ranged from 0.24 to 0.32 in 3 reviewed studies [8]. Furthermore, the range and types of PA vary dramatically, while CRF consists of a single well characterized number.

CRF is a powerful predictor of early mortality independent of PA levels, BMI, or other risk factors [9, 10]. CRF also varies largely within sedentary populations [11]. For instance in a population of 1707 men aged 20–49 the difference between 20th and 80th percentile is more than 30% (36.8–48.5 mL/kg/min) [11]. Likewise the change in CRF to a standardized exercise training program varies largely from −4.7% to +58.0 [12]. While large cross-sectional studies suggest higher CRF are associated with lower levels in biomarkers for metabolic disease other intervention studies show that improvements in CRF are only weakly correlated with improved biomarkers [13].

VO_2max_ in sedentary population of monozygotic twins is highly heritable (77%) even with correction for various anthropometric measures [14]. Similarly, gains in CRF following 20 weeks of endurance exercise training (3 d/wk at 55–75% VO_2max_) are heritable (47%) and highly variable, with some individuals showing no response in VO_2max_ [15]. The variability of the response to endurance training is in part explained by differences in age, sex, race, and initial VO_2max_ [12, 16, 17], an explanation that is not universally found or contributes in totality to the variation of gains in CRF [18].

On the other hand changes in PA can be accomplished through behavioural strategies that are not subject to the same variability that CRF is. Furthermore levels of PA are associated with changes in disease risk. In general, results from the Aerobics Center Longitudinal Study suggest that within groups of individuals who have similar CRF, overall health (cardiovascular health and cancer) is better in individuals with higher PA [6]. One specific example is that PA, independent of CRF, is atheroprotective by improving lipoprotein subclass distribution, postprandial lipoprotein metabolism, inflammation, and endothelial function [19–23]. Conversely, physical inactivity increases the relative risk for at least 35 pathological and clinical conditions [1]. Remarkably, physical inactivity as defined by sitting time is a risk factor for premature death, a number of chronic diseases and pathologies [24]. For example, 20 days of bed rest [25] or 14 days of reduced step count (and thus increased sitting) [26] reduce CRF 27% and 7%, respectively. However, it remains difficult to isolate the independent roles of PA, inactivity, and CRF in health parameters as most studies are not designed to explicitly control for CRF, while changing just PA.

In the present study, we hypothesized that PA would improve metabolic fitness independent of CRF. We therefore sought to identify two groups of people who were closely matched with regard to age, gender, and CRF, but who differed markedly with regard to their PA level. We recruited an endurance-trained group (consisting of recreational marathon runners) and a control group, matched for BMI, age, and gender, and tested various metabolic health parameters (blood lipids, glucose tolerance, and body composition) as our primary outcome. The endurance group fulfilled at least one of two inclusion criteria: (1) they had had an average training volume of at least 50 kilometers per a week for at least the past 5 years or (2) they completed at least 10 marathons within the past 5 years including 2 within the last 14 months. Because of the known variability in CRF it was our aim to compare a subgroup (runners versus controls) with similar-VO_2max_ (similar-VO_2max_) and a subgroup (runners versus controls) with different-VO_2max_ (different-VO_2max_).

## 2. Methods

### 2.1. Recruitment of Subjects

Subjects were recruited through newspaper adverts and emails listserves in 2008-2009 from the greater Copenhagen metro area. Marathon runners fulfilled at least one of two inclusion criteria: (1) having an average training volume of at least 50 kilometers per week for at least the past 5 years, as determined by questionnaire or (2) having ran at least 10 marathons in the past 5 years including 2 within the last 14 months. After a marathon runner had completed the physiological testing a sedentary subject was recruited to match the BMI, age, and sex of the marathon runner. Sedentary subjects were limited to individuals who did not obtain more than 1 hour of structured exercise per week. Exclusion criteria prior to inclusion in the study for both groups included chronic diseases, pregnancy within the last 3 months, abuse of alcohol, use of cigarettes, or use of performance enhancing drugs. In total, 31 pairs of subjects were recruited. No subjects were excluded after testing. The purpose of the study, possible risks, and discomforts were explained to the subjects before written consent was obtained. The study was approved by the Local Ethical Committee of Copenhagen and Frederiksberg and was in accordance with the* Declaration of Helsinki*.

Subjects underwent two days of testing. On day 1, subjects arrived to the lab in a fasted condition and underwent a physical examination during which blood pressure, resting heart rate (HR), and blood were taken for standard laboratory measurements including blood lipids.

Following the examination subjects underwent a muscle biopsy. Briefly, muscle biopsies from vastus lateralis were obtained at rest using the percutaneous Bergstrom needle method with suction under local anaesthesia, using 3–5 mL of 20 mg mL^−1^ lidocaine (SAD, Denmark Copenhagen). Muscle tissue was immediately frozen in liquid nitrogen and stored at −80°C until further analysis. Following the muscle biopsy, subjects underwent a dual-energy X-ray absorptiometry (DEXA) measurement and an oral glucose tolerance test, which was administered at ~10:00 AM.

### 2.2. Separation of Subjects into “Similar-VO_2max_” and “Different-VO_2max_”

After all subjects had undergone the standard testing, each runner-sedentary pair of subjects was ranked by their relative difference in VO_2max_ (mL/kg/min). Runners VO_2max_ ranged from −5% to 64% higher than their sedentary pair. We separated the cohort into thirds and focused our analysis on the 10 pairs of marathon runner and sedentary controls with closest VO_2max_ (−5% to 15%, mean 5% higher, and absolute difference 2.2 mL/kg/min) and the 10 pairs with the largest difference in VO_2max_ (27% to 64%, mean of 44% high, and absolute difference 17.3 mL/kg/min). We refer to these two groups as similar-VO_2max_ and different-VO_2max_, respectively.

### 2.3. Body Composition

Whole body fat and fat-free tissue mass measurements were performed using a dual-energy X-ray absorptiometry (DXA) scanner (Lunar Prodigy, GE Healthcare, WI, Madison USA, software v. 8.8).

### 2.4. Oral Glucose Tolerance Test

Subjects underwent a three-hour oral glucose tolerance test (OGTT). Within 2 minutes, the subject ingested a drink containing 75 g glucose (Dextrose Anhydre, Roquette Freres, France) dissolved in 300 mL of water. Venous blood samples were collected from an antecubital venous catheter before and 10, 20, 30, 60, 90, 120, 150, and 180 minutes after ingestion of the solution. Blood was drawn into tubes (Vacuette, Serum clot activator and Sodium Fluoride/Potassium Oxalate, Hettich, Labinstruments APS) for determination of glucose and insulin to each time point. Blood samples were analyzed for standard biochemical measurements by the biochemical department of Rigshospitalet.

### 2.5. Exercise Testing

The treadmill (Runrace, Technogym, Italy) test consisted of a lactate threshold and maximal oxygen consumption (VO_2max_) portion, which took less than 20 minutes. Indirect calorimetry measurements were collected throughout the test (Quark b2, CosMed, Rome, Italy). For marathon runners the test began at 3 km·h^−1^ slower than their current marathon pace. The controls started at 6-7 km·h^−1^. Every third minute we increased the speed 1 km·h^−1^, the third minute of which VO_2_ was averaged until lactate threshold was reached. After each stage subjects stepped off the treadmill for the 15 s required to obtain a blood sample, after which they began the next stage while the measurement was conducted. Blood samples were collected within 15 sec of the end of the stage by wiping sweat, ethanol cleaning, and drying of a nonlanced finger. Lactate was measured by a handhold lactate device (Lactate Scout, EKF Senslab GmbH, Leipzig, Germany), which requires 0.5 *μ*L whole blood and 10 seconds for a measurement. The workload at which the concentration of blood lactate reached 4.0 mmol/mL or began to increase exponentially was selected as lactate threshold. We performed posttest analysis to ensure that the stage at which lactate threshold was reached showed agreement with the ventilatory threshold.

Without a rest subjects began the VO_2max_ test at the speed lactate threshold was reached. The speed was increased 1 km·h^−1^ every minute until the participant was unable to keep up with speed and/or increasing the workload no longer increased VO_2_ (mL/L). All subjects reached exhaustion or plateaued VO_2max_ and had an RER > 1.10. Heart rate was recorded during the whole test.

### 2.6. Immunoblotting

Immunoblots were completed as previously reported [27]. Briefly, skeletal muscle biopsies were weighed and homogenized using a Tissuelyser (Qiagen) (50 mM Tris·HCl, 150 mM NaCl, 1 mM EDTA, 1 mM EGTA, 50 mM NaF, 5 mM NaP, and 0.2% Ipegal-CA-630) supplemented with complete protease inhibitor cocktail (Roche) and phosphatase inhibitors (Sigma). Protein concentrations were measured with the Bradford assay [28]. Equal amounts of proteins were subjected to SDS-PAGE using Invitrogen 8% precast gels and an I-blot dry transfer machine according to the manufacturer's instructions. Each GEL contained either the different-VO_2max_ group or the similar-VO_2max_ group with pairs loaded in adjacent lanes. 30 *μ*g of protein was loaded in each well. Polyvinylidene fluoride membranes were probed with primary antibodies at the following concentrations: MnSOD (#06-984; Upstate) 1 : 2000, GPX1 (number 3206; Cell Signaling) 1 : 5000, HSP72 (SPA-810F; Gentaur) 1 : 1000, COXIV-3E11 (number 4850; Cell Signaling) 1 : 2000, GLUT4 (PA1-1065; Thermo Fischer) 1 : 1000, VEGF (sc-152; Santa Cruz) 1 : 500, and MHCIIa (number 3403; Cell Signaling) Detection of primary antibodies was performed using either a mouse (Pierce) or rabbit (Dako) peroxidase-conjugated IgG, and protein signals were visualized using FEMTO-enhanced chemiluminescence and a Bio-Rad Chemidoc XRS imager. Equal protein loading and transfer was verified by beta-tubulin signal and total lane reactive brown signal, which stains for total protein. Quantification of the immunoblots was done using Image J (National Institutes of Health, Bethesda, MD, http://rsb.info.nih.gov/ij/) and corrected for total signal on each blot to correct across blots.

### 2.7. Statistics

All statistics were performed in Graph Pad (version 5.00 for Windows, GraphPad Software, San Diego, California, USA, http://www.graphpad.com/). Unless noted in the text, a two-way ANOVA with marathon runner/control and similar-VO_2max_/different-VO_2max_ as the two factors was performed. If either factor showed significance post hoc analysis was done by Bonferroni correction with significance set at *P* < 0.05.

## 3. Results

### 3.1. Matching of Healthy Controls and Marathon Runners

A total of 40 subjects, 20 runners, and 20 sedentary runners were included for analysis to take advantage of similarities or differences in VO_2max_. The subjects were further divided into similar-VO_2max_ (*n* = 10 for runners and sedentary) and different-VO_2max_ (*n* = 10 for runners and sedentary). Basic anthropometrics can be found in Table 1, indicating that the experimental design resulted in well matched subjects by age (*P* = 0.97 for interaction) and body weight (*P* = 0.76 for interaction). The similar-VO_2max_ group runners and sedentary subjects had similar body fat percentages (*t*-test), while the different-VO_2max_ group runners had significantly lower body fat than the sedentary group.

### 3.2. Separation of Paired Subjects Based on Their VO_2max_ Similarity

**Figure 1:**
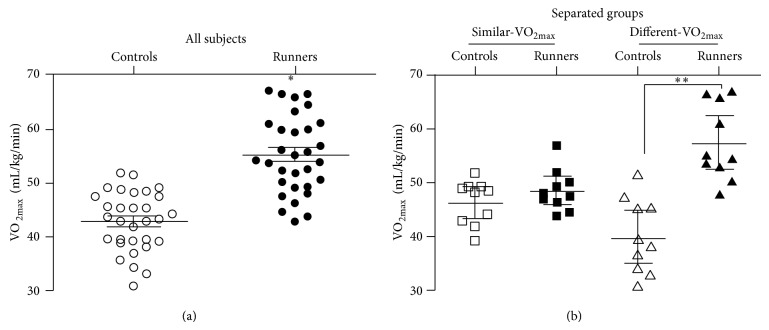
VO_2max_ (mL/kg/min) was determined in marathon runners and controls who were individually matched for age, BMI, and gender with one runner. Runners as a group had a significantly higher VO_2max_ than their sedentary paired control (Panel (a)) (*n* = 31/group, *P* < 0.0001, *t*-test). When we stratified the pairs by difference in VO_2max_ between each runner and their control the 10 pairs with the most similar-VO_2max_ did not significantly differ in VO_2max_ (Panel (b)) (*n* = 10, *P* = 0.22, *t*-test). In 10 pairs with the most different-VO_2max_ the runners had a significantly higher VO_2max_ (*n* = 10, *P* < 0.0001, *t*-test).

As expected runners as a group had a higher VO_2max_ than healthy controls (Figure 1, *P* < 0.0001). In ranking VO_2max_ difference between one marathon runners and one sedentary subject the 31 differences ranged from −5% to 64%, with one-third of the pairs showing a 10% or less difference in VO_2max_. In this subgroup, which we call similar-VO_2max_, runners had on average only a 5.2% higher VO_2max_ (range of −5% to 15% for runners/controls, Figure 1, *P* = 0.22 by *t*-test). The similar-VO_2max_ group allowed us to compare a group of individuals with the same CRF, but different levels of PA. At the other extreme in the 10 pairs of subjects with the largest difference in VO_2max_, which we call different-VO_2max_, runners had on average a 43% higher VO_2max_ (range of 27%–64% higher, Figure 1, *P* < 0.001). The different-VO_2max_ subgroup allowed us to examine the degree and magnitude to which high CRF and high PA were associated with better metabolic health.

### 3.3. Runners Metabolic Profile Was Improved Relative to Healthy Controls

**Figure 2:**
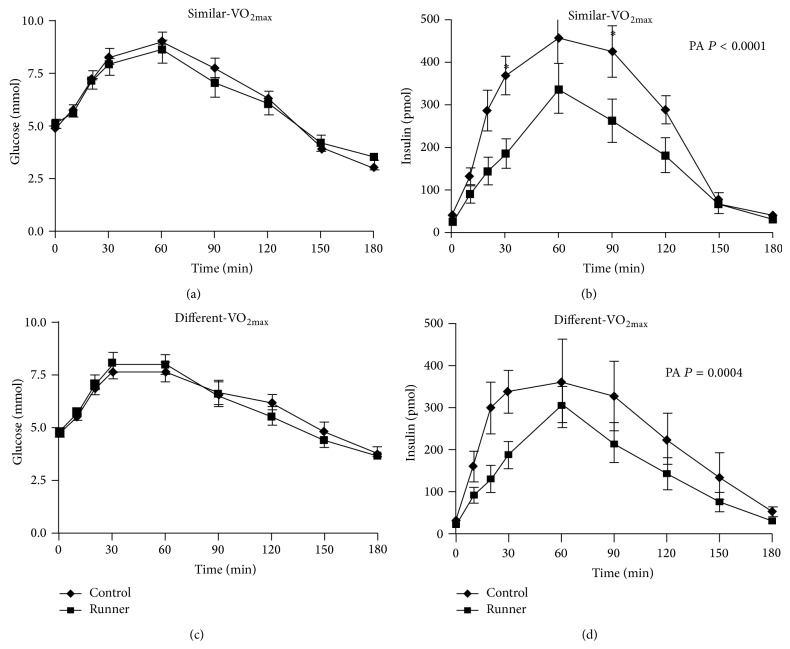
Oral glucose tolerance tests were conducted on marathon runners and sedentary paired controls with similar-VO_2max_ ((a, b),  *n* = 10) and with different-VO_2max_ ((c, d), *n* = 10). While no differences in plasma glucose were seen between runners and sedentary controls regardless of VO_2max_ stratification, runners regardless of VO_2max_ stratification had a significantly lower insulin response (*P* < 0.0005 for both groups, One-Way Repeated Measures ANOVA).

To assess the overall metabolic health of the subjects, blood lipids and oral glucose tolerance tests were conducted. Fasting levels of insulin, total cholesterol, LDL, and triglycerides were all significantly lower, and HDL was significantly higher in the runners versus controls (Table 1). Similarly, the insulin response during an OGTT was significantly lower in both the similar-VO_2max_ and different-VO_2max_ (Figures 2(b) and 2(d)), while the glucose response to an OGTT did not differ (Figures 2(a) and 2(c)). Together these data indicate that in subjects matched for CRF high levels of PA are necessary for improved metabolic risk factors.

### 3.4. Runners Markers of Performance Are Better Than Healthy Controls Despite Similar-VO_2max_

VO_2max_ is not necessarily a predictor of performance so we examined additional markers of exercise performance to see whether runners perform better regardless of their VO_2max_. The velocity at VO_2max_ and speed at lactate threshold was higher in the runners from both the similar-VO_2max_ and different-VO_2max_ groups (Table 2). What may account for the higher velocity at VO_2max_, but not absolute VO_2max_ in the runners from the similar-VO_2max_ group was a difference in running economy. The similar-VO_2max_ group, but not the different-VO_2max_, uses less relative oxygen at a given speed compared to their matched sedentary control group.

### 3.5. Runners Skeletal Muscle Has Higher Antioxidant and Oxidative Enzyme Content Relative to Healthy Controls

**Figure 3:**
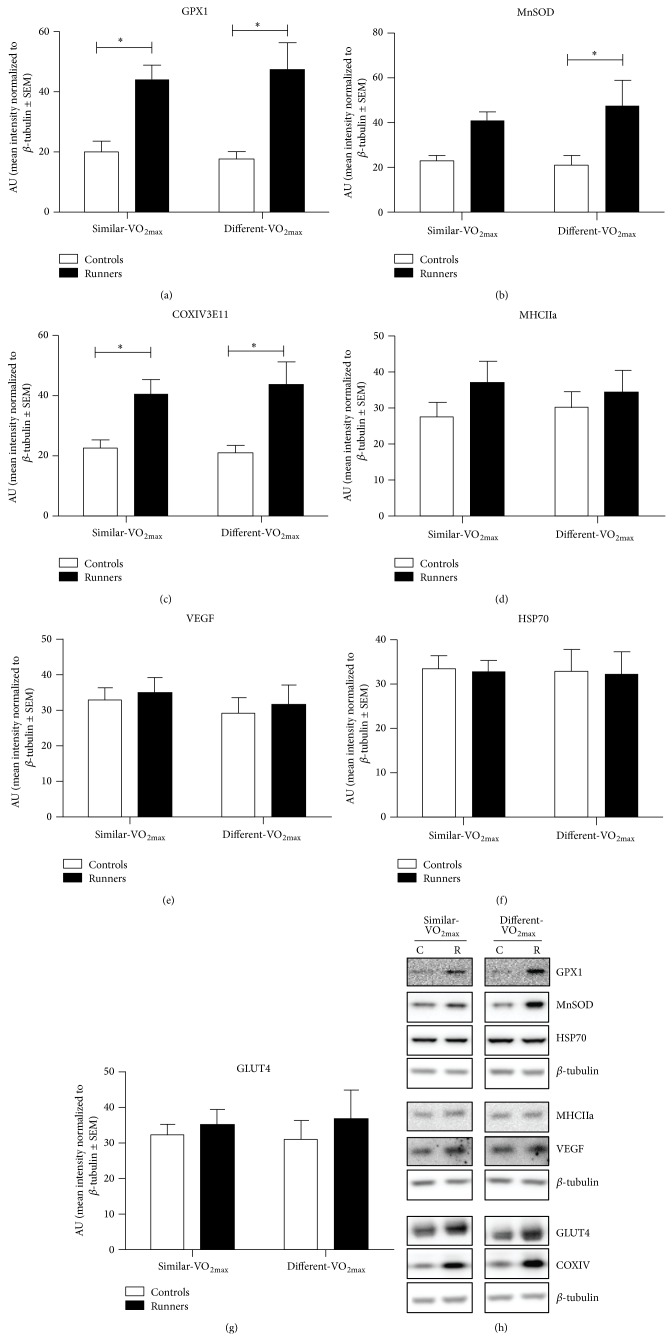
Protein levels as measured by western blot of traditional training markers in runners (solid bars) or pair sedentary controls (open bars) in resting vastus lateralis biopsies. Glutathione peroxidase 1 (a) and manganese superoxide dismutase (MnSOD) (b) were significantly higher in runners (*n* = 10, *P* < 0.001, 2-way ANOVA). Mitochondrial complex IV (COXIV) (c) was significantly higher in runners (*n* = 10, *P* < 0.001, 2-way ANOVA). Post hoc significance as determined by Bonferroni corrected *t*-test is indicated by a ^*^(*P* < 0.05). Myosin heavy chain IIa (d), vascular endothelial growth factor (VEGF) (e), heat shock protein 70 (HSP70) (f), nor glucose transporter 4 (GLUT4) (g) did not differ between groups. Proteins of interest are normalized to *β*-tubulin as a loading control that did not differ between groups. Representative blots are shown in (h), with C indicating control and R indicating runner.

In addition to systemic measures of exercise capacity such as VO_2max_, velocity and VO_2max_, and lactate threshold we wanted to examine whether skeletal muscle markers for antioxidant and mitochondrial content were higher regardless of VO_2max_ in vastus lateralis skeletal muscle biopsies of runners (Figure 3). The skeletal muscle of marathon runners had significantly higher protein content of the antioxidant enzymes glutathione peroxidase 1 (GPX1) and mitochondrial superoxide dismutase (mnSOD), but not heat shock protein 72 (HSP70) (Figure 3). The difference in GPX1 (Figure 3(a)) was higher in runners from both the similar-VO_2max_ and different-VO_2max_ group. Similarly, cytochrome oxidase subunit 4 (COXIV), a mitochondrial enzyme in electron transport chain, was higher in skeletal muscle from the runners in both the similar-VO_2max_ and different-VO_2max_ group (Figure 3(f)). However, runners and sedentary subjects had no differences in protein content glucose transporter 4 (Figure 3(g)), myosin heavy chain isoform IIa (Figure 3(d)), or vascular endothelial growth factor (Figure 3(e)). Thus, high levels of PA, rather than whole body VO_2max_, are associated with higher levels of specific markers of antioxidant capacity and mitochondrial content in skeletal muscle.

## 4. Discussion

The present case-control study clearly shows that a high amount of physical activity (PA) is associated with benefits on metabolic health parameters independent of cardiorespiratory fitness (CRF). Conversely a higher level of CRF without high levels of PA was associated with lower levels of metabolic fitness. Our measures of metabolic fitness included improved blood lipid profile, lower insulin response to an OGTT, and increased skeletal muscle mitochondrial markers. Furthermore, runners within the similar-VO_2max_ had superior exercise performance as measured by velocity at VO_2max_, lactate threshold, and submaximal running economy.

It is well accepted that PA and CRF reduce the risk of cardiovascular disease, diabetes, and all-cause mortality [29–31]. However, the relative contributions of PA and CRF to the reduction in risk are less well known and few studies have been designed to pre hoc separate the effects of PA and CRF from each other. Still analyses of larger cohorts have in cross-sectional and longitudinal studies attempted to separate the effects of CRF and PA on health. For instance, with regard to mortality, the associations of PA and CRF were examined separately and in combination in a cohort of relatively healthy 20–82-year-old men and women [32]. Subjects with high levels CRF had lower mortality, while no association between PA and mortality was found. Conversely, data from another study showed higher levels of both PA and CRF associated with reduced risk factors for cardiovascular disease in a cohort of randomly selected 20–65-year-old Swedish men and women [33]. Furthermore, in attempt to separate the effects of CRF and PA, the author's analysis showed that subjects with a low CRF, but who were physically activity, had a 50% reduction in risk factors. However, the design of the study only groups subjects as only fit/unfit active/nonactive, which is a less robust method to match groups as we employed in the current study. While useful for large cohorts the dichotomization of groups by fit/unfit and active/nonactive may not best represent the complex interaction between CRF and PA.

The finding that the highly PA marathon runners had a more beneficial lipid profile than controls, independent of CRF, is in accordance with previous findings in “at risk groups” [34]. In a classic randomized-controlled trial by Kraus et al. [20] they determined the effect of the amount and intensity of training in 111 sedentary, overweight men and women with mild-to-moderate dyslipidemia. The study design randomly assigned subjects to a control group (no exercise training) or to 8 months of physical training entailing either high-amount-high-intensity exercise (32 km jogging/week at 65–80% of peak oxygen uptake (VO_2max_)), low-amount-high-intensity exercise (19 km jogging/week at 65–80% percent of VO_2max_), or low-amount-moderate-intensity exercise (19 km walking/week at 40–55% of VO_2max_). Regardless of which exercise group they were assigned to all subjects showed improved lipoprotein profile (lower triglycerides, lower vLDL, and lower vLDL particle size) relative to the control group. However, which exercise group subjects were assigned to did make a difference in whether they improved CRF. The group that performed low-amount-high-intensity exercise improved CRF more than the low-amount-low-intensity group. Thus, both groups improved lipid profiles to a similar degree but improved CRF to a different level. In healthy subjects a change in PA without a change in CRF can lead to improved metabolic fitness. For instance, subjects who skied an average of 342 min/day on a 32 day cross country skiing trip across Greenland had a decrease in maximal CRF but still had an improvement in circulating lipoproteins [35]. While both of these studies were interventions lasting less than 9 months, we were interested in whether a similar finding would occur in long-term runners versus long-term sedentary subjects. Indeed we found that sedentary and runners with similar levels of CRF had different lipoprotein and insulin responses to an OGTT test. One potential explanation for this separation in CRF and metabolic health is the type of exercise the marathon runners do. Many of the subjects perform only low-intensity long duration types of exercise. This is in agreement with Houmard et al. [36] who showed that insulin sensitivity was improved more by an exercise training programme with high volume than high intensity. Insulin sensitivity is highly dependent on skeletal muscle specific adaptations, which may differ in response to PA or exercise training that increases CRF.

Some types of PA, such as low intensity or limited to small amount of tissue, may lead to significant local changes important for metabolic health without improved whole body CRF. Adaptation specific to the working muscle is illustrated in a number of studies on one-legged exercise training, which utilize a small fraction of whole body muscle mass result in important local adaptations in trained muscle such as increased capillarization, muscle glycogen content, mitochondrial activity, insulin sensitivity, and transcription of metabolic genes [37–40]. Vollaard et al. [41] find these local changes in muscle metabolism are associated with changes in maximal work capacity more so than VO_2max_. Furthermore, an increase in VO_2max_ with a PA intervention is not even necessary to improve muscle mitochondrial content. For instance, despite not improving CRF cross country, skiers sill improved skeletal muscle mitochondrial [35]. Similarly, in our study it was the high levels of PA, and not a high CRF, that was associated with higher protein levels of the mitochondrial protein COXIV and antioxidant enzyme mnSOD. The exact physiological, genetic, and environmental contributions that determine individuals VO_2max_ vary tremendously and are not completely known.

We were surprised that so many (one-third) of the marathon runners did not have substantially higher VO_2max_ than their sedentary match subject. A number of twin studies (reviewed in [1]) have determined a typical genetic contribution for the baseline physical fitness characteristics and responses to standardized aerobic and strength training programs of approximately 50%. The largest single study examining genetic and exercise training interactions with health outcomes is the HERITAGE Family Study [16], which suggested significant genetic variation in the interaction between VO_2max_ and risk factors such as resting systolic blood pressure, fasting plasma HDL-cholesterol, triglycerides, and insulin. The HERITAGE study showed that between 8% and 13% of exercise intervention participants have worse off risk factors following 20 weeks to 6 months of exercise training programs [42]. Some specific genes that may contribute to these differences in response to exercise have been identified already. An ongoing study listed in 2005 that 165 autosomal gene entries were found to alter physical performance and health parameters, some of which are only present in response to PA, such as variants in AKT [43], perilipin [44], and FTO [45]. However, the HERITAGE study did not examine what proportion of the inactive groups developed worse risk factors. Other studies which include an inactive control group show worsening of 12 different risk factors including 3 of the 4 examined above (blood pressure was not measured) following 6 months of inactivity [46]. Our current cross-sectional study design only allows us to make association and future studies that trained subjects over a period of time or followed the same subjects over a number of years could provide information about the relative contribution of environment and genetics.

We believe our study has distinctions over previous studies. First is that we selected runners that have been running for at least 5 years in a fairly consistent manner, either completing two marathons per a year or having averaged more than 50 km/week over the past 5 years. This is substantially more exercise for a much longer duration than the large clinical intervention studies such as the HERITAGE and STRRIDE studies [15, 46]. Second, we recruited everyday athletes rather than elites, which have been studied at older ages previously. For instance, Trappe et al. [47] examined the performance abilities of elite octotarian lifelong athletes versus healthy untrained men. Octotarian athletes had VO_2max_ and max workloads that were 80% and 51% higher than a matched sedentary group. While our subjects spanned a much larger age range the subjects most runners had significantly higher VO_2max_ and max workload (running speed). Excluding the runners in the similar-VO_2max_ group (*r* ^2^ = 0.04) VO_2max_ and max workload were highly correlated (*r* ^2^ = 0.48–0.80), a relationship that is also present in octotarian athletes and sedentary controls. In general the runners in the similar-VO_2max_ group had higher max workloads than their VO_2max_ would predict. This discrepancy between max workload and VO_2max_ is likely due to the superior running economy it the similar-VO_2max_ group compared to their sedentary controls. Together the data suggests that multiple types of physiological adaptations may occur to result in an improved work capacity following years of high levels of PA.

There are a number of limitations in the present study that are worth discussing. First, although within our similar-VO_2max_ group there was no statistical difference between the runners and the sedentary group there was a 5% relative (2.2 mL/kg/min absolute) higher-VO_2max_ that may have contributed to some of the metabolic benefits seen in the runners. However, runners in both the similar-VO_2max_ and different-VO_2max_ had similar improvements in metabolic fitness, arguing against the relative difference in VO_2max_ being important for metabolic health. Second, a larger subject population number would have allowed for more advanced statistical analysis that could model the variables as continuous and more precisely determine the relative contribution of VO_2max_ to different metabolic phenotypes. However, given our difficult inclusion criteria for the runner group and difficulty in finding BMI and age matched sedentary controls, it would still remain difficult to recruit hundreds of subjects. Similarly because the training that the runners did was not controlled there are likely a number of individual differences within the runner group that may contribute positively or negatively to the findings seen. Finally, while we observed improved metabolic health of the runners in our group, the metabolic health of the sedentary group remained within clinically normal ranges. However, even within the normal range of clinical variables, small variations in lipoproteins [48, 49] and blood pressure [50] are associated with varied outcomes and risks for cardiovascular disease and death.

In summary the present cross-sectional case-control study suggests that a high amount of PA independent of CRF is associated with positive benefits on a number of metabolic health parameters as well as markers of performance.

## Figures and Tables

**Table 1:** 

	*n*	Age	BMI	Fat-free mass (kg)	Fat %	Fasting glucose (mM)	Fasting insulin (pmol)	Total cholesterol (mM)	HDL (mM)	LDL (mM)	TAGs (mM)
		Similar-VO_2max⁡_									
Controls	10 (9 M, 1 F)	46.8 ± 2.9 (32–61)	23.6 ± 0.5 (21.0–26.9)	60.5 ± 3.2	19.0 ± 1.9 (10.6–27.6)	5.9 ± 0.1	35 ± 5	5.5 ± 0.3	1.5 ± 0.1	3.5 ± 0.2	1.13 ± 0.17
Runners	10 (9 M, 1 F)	46.2 ± 2.7 (31–60)	23.8 ± 0.6 (21.7–25.7)	58.4 ± 2.1	19.1 ± 2.2^#^ (8.3–26.7)	6.1 ± 0.1	29 ± 5^#^	4.9 ± 0.3^#^	1.7 ± 0.2^#^	2.9 ± 0.3^#^	0.90 ± 0.15^#^

		Different-VO_2max⁡_									
Controls	10 (7 M, 3 F)	42.5 ± 3.1 (32–64)	23.4 ± 0.6 (20.8–26.1)	57.7 ± 3	26.7 ± 2.2 (17.0–36.6)	6.2 ± 0.2	38 ± 5	5.7 ± 0.4	1.5 ± 0.1	3.4 ± 0.3	1.28 ± 0.23
Runners	10 (7 M, 3 F)	42.4 ± 3.1 (29–62)	23.0 ± 0.5 (21.4–27.4)	60.3 ± 2.7	14.9 ± 1.9^#∧^ (6.9–24.0)	5.8 ± 0.2	25 ± 4^#^	4.6 ± 0.2^#∧^	1.9 ± 0.1^#^	2.5 ± 0.2^#^	0.76 ± 0.08^#^

All data presented as mean ± SEM, with the range of values within parentheses. # indicates significant effect of marathon running. ∧ indicates post hoc difference in runner versus control within either similar or divergent group. M = males, F = females, BMI = body mass index, HDL = high density lipoproteins, LDL = low density lipoproteins, and TAGs = triglycerides.

**Table 2:** 

	VO_2max⁡_ (mL/kg/min)	VO_2max⁡_ (mL/kg FFM/min)	Predicted VO_2max⁡_	*v* VO_2max⁡_ (k/h)	Lactate threshold (k/h)	RE (mL O_2_/kg/km)	Resting HR (bpm)
Similar-VO_2max⁡_							
Controls	46.3 ± 1.6	58.2 ± 1.6	39.2 ± 2.4	13.2 ± 0.6	10.1 ± 0.5	234.2 ± 6.9	58 ± 2
Runners	48.5 ± 1.2^#^	62.5 ± 1.4^#^	51.6 ± 4.4^#∧^	15.7 ± 0.5^#∧^	13.0 ± 0.6^#∧^	205.7 ± 8.8^#∧^	50 ± 3^#^

Different-VO_2max⁡_							
Controls	40.2 ± 2.2	54.2 ± 1.8	36.9 ± 3.5	11.8 ± 0.6	8.2 ± 0.5	224.4 ± 3.5	59 ± 3
Runners	57.5 ± 2.2^#∧^	69.9 ± 2.2^#∧^	57.3 ± 4.0^#∧^	17.2 ± 0.9^#∧^	14.5 ± 0.5^#∧^	210.8 ± 5.1^#∧^	47 ± 3^#∧^

All data presented as mean ± SEM. # indicates significant effect of marathon running. ∧ indicates post hoc difference in runner versus control within either similar or divergent group. VO_2max⁡_ = ventilation of maximum oxygen consumption, FFM = fat-free mass, *v* VO_2max⁡_ = velocity at VO_2max⁡_, k/h = kilometers per hour, RE = running economy, HR = heart rate, and bpm = beats per a minute.
